# The neurodevelopmental basis of schizophrenia: clinical clues from craniofacial dysmorphology in northwest Ethiopia, 2020

**DOI:** 10.1186/s12868-021-00663-y

**Published:** 2021-09-29

**Authors:** Binalfew Tsehay, Girma Seyoum

**Affiliations:** 1 Department of Biomedical Sciences, Debre Markos University, Debre Markos, Ethiopia; 2 Department of Anatomy, Addis Ababa University, Addis Ababa, Ethiopia

**Keywords:** Craniofacial dysmorphology, Schizophrenia, Neurodevelopment, Ethiopia

## Abstract

**Background:**

The neurodevelopmental speculation of schizophrenia states that the pathogenesis of schizophrenia starts with early fetal or neonatal neurocraniofacial development rather than youthful adulthood when manic signs and symptoms are evident. However, there is no direct evidence of a pre-or peri-natal lesion associated with schizophrenia, rather indirect evidence of impaired development can be seen in macroscopic anatomical variations as well as microscopic immunohistochemical anomalies. One approach to studying neurodevelopmental disturbances among schizophrenic patients is somatic physical evidence or neurodevelopmental markers. Thus Our study aimed to assess the neurodevelopmental basis of schizophrenia clinical clues from anthropometric assessment of craniofacial dysmorphology among schizophrenic patients in North West Ethiopia 2019–2020.

**Method:**

Institutional-based comparative cross-sectional study design was conducted in Debre Markos comprehensive specialized hospitals in 190 schizophrenic patients, 190 1st-degree relatives, and 190 healthy controls. Data were collected using standard methods, entered into EpiData version 3.1, and exports to SPSS version 24 for analysis. Descriptive data were analyzed using descriptive statistics. Welch ANOVA and post hoc comparison, a Games-Howell test, were conducted. Significance was set at a p-value of α = 0.05. Read back analysis was also conducted for the conclusion.

**Results:**

Five hundred seventy study samples, male 375(65.8%), and female 195 (34.2%), were included in this study. The Games-Howell test revealed that the coronal arc length and sagittal arc length among schizophrenic patients were statistically significantly longer than the healthy controls (p < 0.006; p < 0.001, respectively). However, the difference between schizophrenic and healthy control regarding head circumference was marginally significant (p = 0.056). Schizophrenic patients had a significantly shorter total facial height (p < 0.001) and upper facial height (p < 0.001) than healthy controls. Regarding facial depth, schizophrenic patients had significantly shallow upper facial depth (p < 0.001), middle facial depth (p = 0.046), and lower facial depth (p < 0.001).

**Conclusion:**

our finding indicated indirect evidence for disturbed craniofacial development in schizophrenia patients, and close and read back analysis of the result supported the neurodevelopmental basis of disease.

**Supplementary Information:**

The online version contains supplementary material available at 10.1186/s12868-021-00663-y.

## Introduction

### Background

The etiology and pathophysiology of schizophrenia remain poorly understood even though the availability of advanced diagnostic modalities. The neurodevelopmental theory of schizophrenia, the widely accepted theory, states that the pathogenesis of schizophrenia starts with early intrauterine fetal or neonatal neurodevelopment rather than during adulthood when manic signs become apparent [1–4]. The developmental disturbances during early life specifically during the period of embryogenesis underlie the structural and functional changes in the brain and the craniofacial region [5, 6].

The researchers on the various neurodevelopmental theories believe that the primary pathogenic defect of schizophrenia occurred during the early neurocraniofacial development with an essentially static process of the causative agents. The behavioral consequences of the patients i.e., manic signs and symptoms remain relatively latent until puberty [7].

Generally, the craniofacial region and the brain develop from the same ectodermal tissue during the 5–13th weeks of gestation [5, 8, 9]. If a disturbance occurs during this time, the process of dysmorphology may leave “fossil marks” in the craniofacial regions, which are clinically dormant until after puberty, and the corresponding parts of the brain. According to the neurodevelopmental theory of schizophrenia, neurodevelopmental disorders are often concentrated in the craniofacial region of schizophrenic patients [5, 10, 11].

If schizophrenia is a neurodevelopmental disorder, individuals with schizophrenia should show an increased rate of craniofacial dysmorphic features which are clinically and cosmetically insignificant [12–14]. However these features can be used as markers of disturbed development or clues in the diagnosis of a specific pattern of disease; may provide indirect clues to brain pathology, and could provide clues to the process of schizophrenia as they reflect adverse events during critical periods of development [15].

There is no direct evidence of a pre-or peri-natal lesion associated with schizophrenia, but indirect evidence of impaired development can be seen in macroscopic anatomical variations as well as microscopic immunohistochemical anomalies. Somatic physical evidence or neurodevelopmental markers, measurable in adults, may reflect abnormal neurodevelopmental processes that occurred before or shortly after birth [16, 17]. Craniofacial dysmorphology may provide another clue to the neurodevelopmental processes in schizophrenia since cerebral morphogenesis is very closely related to craniofacial morphogenesis and is shaped by shared morphogenetic forces [18, 19].

Embryologically inferred measures of craniofacial dysmorphology in psychiatric diseases like schizophrenia may give an understanding of their fundamental etiologies and pathogenesis among patients in Ethiopia. Besides, based on destiny outline correspondences, the pattern of anomalies inside embryonic primordia may predict loci of brain maldevelopment, which can be assessed in brain imaging studies [20]. An early Assessment of craniofacial dysmorphology in the population could be used as a tool among diagnostic criteria in making an early screening of schizophrenia.

## Methods

### Study design and setting

The institutional-based comparative cross-sectional study design was conducted among 190 schizophrenic patients, 190 1st-degree relatives, and 190 healthy controls from December 2019 to June 2020 in Debre Markos referral hospital, Debre Markos. Debre Markos referral hospital and Debre Markos university host a psychiatry center in Debre Markos town that serves the East Gojjam, West Gojjam, and Awi zones.

### Study participants

There were three study groups: The patient group with schizophrenia, the healthy control group who did not have a personal or family history of psychosis and who did not have a current mental illness, first-degree relatives of the patient groups (siblings and parents of patients) in the study and had no schizophrenia themselves.

Selection criteria: Schizophrenic patients and their 1st-degree relatives were recruited from Debre Markos comprehensive specialized hospital psychiatry outpatient department during study periods. The healthy controls were selected from Debre Markos comprehensive specialized hospital and Debre Markos university medical school staffs and students. Exclusion criteria for all study groups included a history of alcoholism or drug addiction, a history of somatic disorder with neurological components, history of head injury, epilepsy, identified craniofacial syndrome, diagnosed organic brain disease, oral-maxillofacial surgery, and schizophrenic patients with aggressive and impulsive behaviors. First-degree relatives with schizoaffective disorder and who met DSM-III-R diagnostic criteria for a psychotic disorder were excluded from the study. Controls were also excluded if they had a first-degree relative with a history of psychosis and psychiatric hospitalization.

### Sample size calculation

The sample size was calculated using Fleiss CC Formula on epi info version 7.0 software; by setting a significance level of 95%, power of 80%, and a case–control ratio of 1:1. The final sample size was 570 (190 Cases and 190 Controls, and 190 1st degree relatives.

### Data collection procedures and quality control

Sociodemographic and psychiatric characteristics of the study sample were collected using a structured interviewer-administered questionnaire. The diagnosis of patient control status was based on the Statistical Manual of Mental Disorders 3rd edition (DSM-III-R) by senior psychiatrists and mental health professionals.

Assessment of craniofacial dysmorphology: anthropometric assessment of craniofacial dysmorphology, measurement tools, positioning of the subject, anthropometric landmarks, and methods of measurement were based on Farkas craniofacial atlas, Anthropometry of the Head, and Face [21]. Craniofacial dimensions were measured unilaterally and bilaterally among schizophrenic patients, their 1st-degree relatives, and healthy control using a digital caliper and tape meter to the nearest 0.1 cm, as distances between standard anthropological landmarks. Standard or rest positions of the head are employed, depending on the requirements for each measurement. Some distance measurements and all inclinations are recorded with the subject’s head in the Frankfort horizontal position. An anthropometric assessment of craniofacial dysmorphology was performed by well-trained assessors. The training was given by senior anatomists specifically regarding anthropometric landmarks based on Farkas craniofacial atlas, Anthropometry of the Head, and Face. The assessors were blind to patient/control status throughout the evaluations. Many landmarks lie directly on the craniofacial surface, and others are identified by their underlying bony structure. (Additional file 1). The following craniofacial measurements were taken from the cranial region: Head length (glabella to opsithocranion); head width (skull base width) (between both tragions); head height (Frankfurt horizontal plane to the vertex); axial arc length (Head circumference); coronal arc length (arc length between both tragions through the vertex); sagittal arc length (arc length between glabella and opsithocranion, through the vertex).

From the facial region: total facial height (from the trichion to the gnathion); the upper facial height (the trichion to the subnasale); trichion to glabella;glabella-subnasale;glabella-stomion;glabella-gnathion;gnathion-nasion, the lower facial height (the subnasale to the gnathion), tragus to subnasale (middle facial depth), tragus to gnathion (lower facial depth), tragus to trichion (upper facial depth) and intercanthal distance (distance between tear ducts) were assessed.

### Variables of the study

The dependent variables were craniofacial dysmorphology among study subjects, whereas sociodemographic characteristics and patient control status were independent variables.

### Definition of variable

#### Schizophrenia

Schizophrenia is a severe mental disorder, diagnosed using Structured Clinical Interviews for DSM-III-R patients.

Craniofacial dysmorphology refers to alternations of ‘normal’ or typical morphology that can be observed and/or measured in a population.

### Data processing and analysis

Data were entered via Epi data version 3.1 and analyzed using SPSS version 24. Descriptive statistics were analyzed and presented in tables. A one-way ANOVA was conducted to compare the means of craniofacial measurements among schizophrenics, their 1st-degree relatives, and healthy control. Normality checks using Levene’s test were carried out for the assumption of one-way ANOVA, and the data violated the homogeneity of variances. Therefore, a Welch ANOVA and alternative post-hoc tests (i.e., a Games-Howell test instead of a Tukey post-hoc test) were conducted. Significance was set at a p-value of α = 0.05. As a result of the bulk of available evidence, consideration of a reverse timeline read-back analysis was conducted in the paper’s discussion section.

### Ethics approval and consent to participate

Written informed consent was obtained from the participants or their families’ patients, and the study was conducted as per the Declaration of Helsinki. The ethical clearance was taken from Debre Markos University, school of medicine Ethical review committee. A permission letter has also been accepted by the hospital.

## Results

### Sociodemographic characteristics and clinical details of the study samples

A total of 570 study samples, male 375(65.8%), and female 195 (34.2%) were included in this study. The mean ages of people with schizophrenia, their 1st-degree relatives, and healthy controls were 35.62 years, 39.97 years, and 33.56 years respectively, with no significant difference between patients and control. 44.73% (85) of schizophrenic patients were females. 93.2% of schizophrenic patients experienced early-onset schizophrenia. 58.4% of schizophrenic patients had a history of illness for not more than 5 years.

### Comparison of anthropometric assessments of craniofacial dysmorphology among study groups

The descriptive statistics showed the mean, standard deviation, and 95% confidence intervals for the dependent variable anthropometrically assessed cranial dimensions (Table 1) and facial dimensions (Table 2) for each separate group.

**Table 1:** Descriptive statistics of anthropometric measurements of cranial dimensions among participants with schizophrenia, 1st-degree relatives, and healthy controls

Cranial anthropometric measurements (cm)	N	Mean (cm)	Std. deviation	95% confidence interval for mean	
				Lower bound	Upper bound
Head length					
Schizophrenic	190	28.02	3.41	27.53	28.51
1st degree relatives	190	27.85	0.82	27.73	27.97
Healthy control	190	28.2	0.84	28.08	28.32
Head width					
Schizophrenic	190	13.47	1.21	13.29	13.64
1st degree relatives	190	13.03	1.03	12.89	13.18
Healthy control	190	13.43	0.91	13.3	13.56
Head height					
Schizophrenic	190	17.74	1.56	17.51	17.96
1st degree relatives	190	17.4	0.99	17.26	17.54
Healthy control	190	17.44	1.36	17.24	17.63
Axial arc length (head circumference)					
Schizophrenic	190	55.25	4.96	54.54	55.96
1st degree relatives	190	55.78	1.65	55.55	56.02
Healthy control	190	56.19	2.59	55.82	56.56
Coronal arc length					
Schizophrenic	190	35.67	2.27	35.34	35.99
1st degree relatives	190	34.81	1.97	34.52	35.09
Healthy control	190	34.88	2.71	34.49	35.27
Sagittal arc length					
Schizophrenic	190	30.84	1.95	30.57	31.12
1st degree relatives	190	30.14	1.91	29.86	30.41
Healthy control	190	29.79	3.09	29.35	30.24

**Table 2:** Descriptive statistics of anthropometric measurements of facial measurements among participants with schizophrenia, 1st-degree relatives and healthy controls

Facial anthropometry measurements	N	Mean (cm)	Std. deviation	95% confidence interval for mean	
				Lower bound	Upper bound
The total facial height					
Schizophrenic	190	17.60	1.64	17.37	17.84
1st degree relatives	190	18.41	1.21	18.24	18.58
Healthy control	190	18.32	1.22	18.14	18.49
Length of the upper facial region					
Schizophrenic	190	11.61	1.32	11.42	11.79
1st degree relatives	190	12.31	.86	12.19	12.44
Healthy control	190	12.41	1.39	12.21	12.61
Trichion to glabella					
Schizophrenic	190	3.99	.81	3.87	4.10
1st degree relatives	190	4.12	0.52	4.04	4.19
Healthy control	190	4.36	0.8	4.26	4.47
Glabella-subnasale					
Schizophrenic	190	7.68	1.27	7.50	7.86
1st degree relatives	190	8.28	1.18	8.11	8.45
Healthy control	190	8.29	1.13	8.13	8.46
Glabella-stomion					
Schizophrenic	190	9.90	1.55	9.67	10.12
1st degree relatives	190	10.61	1.14	10.45	10.78
Healthy control	190	10.42	1.05	10.27	10.57
Glabella-gnathion					
Schizophrenic	190	13.67			
1st degree relatives	190	14.31	1.54	13.45	13.89
Healthy control	190	14.14	0.99	14.17	14.45
Gnathion-nasion					
Schizophrenic	190	10.79	1.0	14.0	14.28
1st degree relatives	190	11.39	1.43	10.59	11.0
Healthy control	190	11.27	1.18	11.22	11.56
The lower facial region					
Schizophrenic	190	6.06	1.11	11.11	11.43
1st degree relatives	190	6.064	1.1	5.90	6.21
Healthy control	190	6.10	0.84	5.94	6.18
Tragus to subnasale (middle facial depth)					
Schizophrenic	190	12.41	1.13	5.94	6.26
1st degree relatives	190	12.5	0.88	12.29	12.54
Healthy control	190	12.61	0.7	12.4	12.60
Tragus to gnathion (lower facial depth)					
Schizophrenic	190	13.61	0.7	12.51	12.71
1st degree relatives	190	13.67	0.81	13.50	13.73
Healthy control	190	13.97	0.75	13.56	13.78
Tragus to trichion (upper facial depth)					
Schizophrenic	190	13.67	0.69	13.87	14.07
1st degree relatives	190	13.85	1	13.53	13.81
Healthy control	190	14.15	0.76	13.74	13.96
Intercanthal distance					
Schizophrenic	190	3.042	0.98	14.01	14.29
1st degree relatives	190	3.122	0.36	2.99	3.09
Healthy control	190	3.074	0.26	3.09	3.16

A Welch ANOVA showed significant difference in mean of the head length Welch’s F (2, 342.2) = 8.399, p < 0.001; head width (p < 0.01); head height (p = 0.041); head circumference (p = 0.045); coronal arc length (p < 0.001) and sagittal arc length (p < 0.001) (Table 3). Similarly, there was a significant difference in mean of the total facial height Welch’s F (2, 372.1) = 16.33, p < 0.001; the upper facial height Welch’s F (2, 358.7) = 22.92, p < 0.001; the upper facial depth Welch’s F (2, 371.6) = 11.7, p < 0.001; the lower facial depth Welch’s F (2, 376.5) = 12.99, p < 0.001) and the intercanthal distance Welch’s F (2, 370.8) = 3.43, p = 0.033) between the study groups (Table 4).

**Table 3:** Robust tests of equality of means of cranial dimensions among study groups

Cranial dimensions in centimetre		Statistic^a^	df1	df2	Sig.
Head length	Welch	8.399	2	342.209	.000
Head width	Welch	10.213	2	373.176	0.000
Head height	Welch	3.220	2	363.324	0.041
head circumference	Welch	3.137	2	333.502	0.045
Coronal arc length	Welch	8.672	2	372.092	0.000
Sagittal arc length	Welch	10.311	2	366.011	0.000

^a^Asymptotically F distributed

**Table 4:** Robust tests of equality of means of facial dimensions among study groups

Facial dimensions		Statistic^a^	df1	df2	Sig.
The total facial height	Welch	16.330	2	372.055	0.000
Length of the upper facial region	Welch	22.923	2	358.745	0.000
Trichion to glabella	Welch	10.794	2	360.153	0.000
Glabella-subnasale	Welch	15.245	2	377.238	0.000
Glabella-stomion	Welch	13.336	2	370.037	0.000
Glabella-gnathion	Welch	11.476	2	367.300	0.000
Gnathion-nasion	Welch	10.512	2	374.061	0.000
The lower facial region	Welch	0.091	2	370.401	0.913
Tragus to subnasale	Welch	2.983	2	374.245	0.052
Tragus to gnathion	Welch	12.992	2	376.517	0.000
Tragus to trichion	Welch	11.670	2	371.639	0.000
Intercanthal distance	Welch	3.433	2	370.791	0.033

^a^Asymptotically F distributed

The Games-Howell, multiple comparisons method, revealed that the coronal arc length and sagittal arc length among schizophrenic patients were statistically significantly longer than the healthy control (p<0.006; p<0.001 respectively). However, the difference between schizophrenic and healthy control regarding head circumference was marginally significant (p =.056). The test also suggested the presence of a significant difference between schizophrenic patients and their 1st-degree relatives regarding head width (p=0.001), head height (p=0.036), coronal arc length (p<0.001), and sagittal arc length (p=0.001) (Table 5). In the same way, schizophrenic patients had a significantly shorter total facial height (p<0.001) and upper facial height (p<0.001) than healthy controls. Regarding facial depth, schizophrenic patients had significantly shallow upper facial depth (p<0.001), middle facial depth (p=.046), and lower facial depth (p<0.001) than healthy controls (Table 6).

**Table 5:** The Games-Howell multiple comparisons table to show specific group differences between schizophrenic patients, 1st-degree relatives, and healthy control

Cranial anthropometric dimensions in (cm)	(I) patient control status of the study sample	(J) patient control status of the study sample	Mean difference (I–J)	Sig.	95% Confidence interval	
					Lower bound	Upper bound
Head length	Schizophrenic	1st degree relatives	0.16947	0.784	− 0.4315	0.7704
		Healthy control	− 0.17921	0.762	− 0.7808	0.4224
Head width	Schizophrenic	1st degree relatives	0.43474^a^	0.001	0.1633	0.7062
		Healthy control	0.03526	0.945	− 0.2240	0.2945
Head height	Schizophrenic	1st degree relatives	0.33316^a^	0.036	0.0174	0.6489
		Healthy control	0.29921	0.116	− 0.0542	0.6526
Head circumference	Schizophrenic	1st degree relatives	− 0.53474	0.338	− 1.4296	0.3601
		Healthy control	− 0.93737	**0.056**	− 1.8942	0.0195
Coronal arc length	Schizophrenic	1st degree relatives	0.86105^a^	0.000	0.3478	1.3743
		Healthy control	0.79000^a^	**0.006**	0.1863	1.3937
Sagittal arc length	Schizophrenic	1st degree relatives	0.70632^a^	0.001	0.2404	1.1722
		Healthy control	1.05053^a^	**0.000**	0.4262	1.6749

^a^The mean difference is significant at the 0.05 level

Bold values indicates significant craniofacial differences between schizophrenic patients and healthy controls

**Table 6:** The Games-Howell multiple comparisons table to show specific group differences of facial dimensions between schizophrenic patients, 1st-degree relatives, and healthy controls

Facial anthropometric dimensions in (cm)	(I) patient control status of the study sample	(J) patient control status of the study sample	Mean difference (I–J)	Std. error	Sig.
The total facial height	Schizophrenic	1st degree relatives	− 0.80947^a^	0.14804	0.000
		Healthy control	− 0.71579^a^	0.14854	**0.000**
Length of the upper facial region	Schizophrenic	1st degree relatives	− 0.70789^a^	0.11420	0.000
		Healthy control	− 0.80684^a^	0.13896	**0.000**
Trichion to glabella	Schizophrenic	1st degree relatives	− 0.12947	0.06950	0.151
		Healthy control	− 0.37211^a^	0.08215	**0.000**
Glabella-subnasale	Schizophrenic	1st degree relatives	− 0.59632^a^	0.12556	0.000
		Healthy control	− 0.61158^a^	0.12334	**0.000**
Glabella-stomion	Schizophrenic	1st degree relatives	− 0.71737^a^	0.13970	0.000
		Healthy control	− 0.52421^a^	0.13590	**0.000**
Glabella-gnathion	Schizophrenic	1st degree relatives	− 0.63737^a^	0.13303	0.000
		Healthy control	− 0.46895^*^	0.13321	**0.001**
Gnathion-nasion	Schizophrenic	1st degree relatives	− 0.59789^a^	0.13438	0.000
		Healthy control	− 0.47368^a^	0.13118	**0.001**
The lower facial region	Schizophrenic	1st degree relatives	− 0.00895	0.10036	0.996
		Healthy control	− 0.04526	0.11430	0.917
Tragus to subnasale	Schizophrenic	1st degree relatives	− 0.08474	0.08192	0.556
		Healthy control	− 0.19474^a^	0.08171	0.046
Tragus to gnathion	Schizophrenic	1st degree relatives	− 0.05842	0.08000	0.746
		Healthy control	− 0.35579^a^	0.07737	**0.000**
Tragus to trichion	Schizophrenic	1st degree relatives	− 0.18411	0.09102	0.108
		Healthy control	− 0.48326^a^	0.10141	**0.000**
Intercanthal distance	Schizophrenic	1st degree relatives	− 0.08053^a^	0.03222	0.034
		Healthy control	− 0.03263	0.03423	0.607

^a^The mean difference is significant at the 0.05 level

Bold values indicates significant craniofacial differences between schizophrenic patients and healthy controls

## Discussion

Embryologically inferred measures of craniofacial dysmorphology in psychiatric diseases like schizophrenia may give an understanding of their fundamental etiologies and pathogenesis. Besides, based on destiny outline correspondences, the pattern of anomalies inside embryonic primordia may predict specific parts of brain maldevelopment, which can be assessed in brain imaging studies [20]. One approach to studying neurodevelopmental disturbances among schizophrenic patients is somatic physical evidence or neurodevelopmental markers or lingering fossil marks. These markers are measurable in adults and reflect abnormal neurodevelopmental processes that occurred before or shortly after birth [16, 17]. This anthropometric approach to the evaluation of dysmorphogenesis is reliable in the accurate assessment of 3-dimensional structures such as the craniofacial region.

In this study, patients with schizophrenia show multiple dysmorphic features of the craniofacial region that readily distinguish them from healthy controls and relatives. The Games-Howell test regarding cranial anthropometric dimensions showed that the coronal arc length and sagittal arc length among schizophrenic patients were statistically significantly longer than the healthy control. However, the difference between schizophrenic and healthy control regarding head circumference was marginally significant.

Similarly, research on morphometric characteristics of craniofacial features in patients with schizophrenia showed significantly increased coronal and Sagittal arc lengths among patients [22]. An increase in the length of the coronal arc in schizophrenia patients in our study represent a wider skull base, which may be a result of alteration in the ossification sequences of the chondrocranium and associated with the expansion of middle cranial fossa (basicranium), and consequently the volume of cerebral temporal lobe [23–25]. Regarding head circumference, our results are consistent with previous reports suggesting cranial size is increased [26] or at least is not decreased in schizophrenia [27, 28].

In the initial stages of embryogenesis, during the 5–13th weeks of gestation, there is a very close relationship between the craniofacial development and the ventral portion of the brain; therefore, maldevelopment in one predisposes the other for malformation [9, 29]. In support of this, the anthropometric analysis showed the presence of maldevelopment of the middle portion of the skull in patients with schizophrenia [9, 10, 30].

Regarding the facial measurements, schizophrenic patients in our study had a significantly shorter total facial height and upper facial height than healthy controls. They also had significantly shallow upper, middle, and lower facial depth. Another research using simple linear anthropometric measurements made with measuring tapes and calipers has shown that schizophrenic patients have more craniofacial disproportionality than healthy control subjects [11, 25, 31].

Recent research has shown that middle cranial fossa size and shape influence the relationship between skull base shape and facial measures. The dimensions of the middle cranial fossa from which the midface attaches and grows forward from the middle cranial fossae are intimately linked to the width of the skull [32–34]. Variations in size and shape of middle cranial fossae may have predictable quantitative effects on many of the craniofacial measures that were found to differentiate those with schizophrenic from controls.

The shape and volume of the endocranial fossa are strongly correlated with the shape and volume of the adjacent parts of the brain which is lodged within the fossae. The middle cranial fossa is closely related to the anterior portion of the temporal lobe [35, 36]. Systematic reviews and meta-analyses of published articles regarding regional brain volume have identified a decreased temporal lobe volumes among schizophrenic patients as compared with their controls [37, 38].

We predict that the anthropometric differences found in the craniofacial region of schizophrenic patients may be as a result of features related to the development of the brain/cranial fossa complex. This can be supported by the presence of significant craniofacial dysmorphology differences between schizophrenics and their first-degree relatives in this study.

A clear understanding of the relation between craniofacial dysmorphology and the risk of schizophrenia and more detailed knowledge of how the developing brain interacts with the neuro basicranial complex may help discover novel candidate exposures and genes related to schizophrenia. Research on how the brain and skull interact during development may provide new ways the understanding schizophrenia.

The consistency of results across multiple studies with ethnic and geographical backgrounds including ours supports the hypothesis that individuals with schizophrenia have increased rates of prenatal developmental disturbances than the controls and their first degree realtives. An assessment of craniofacial dysmorphology in the population could be used as a tool among diagnostic criteria in making an early screening of schizophrenia. Similarly, it can be incorporated to diagnose schizophrenia objectively an addition to subjective analysis.

## Limitation of the study

The absence of imaging modalities like MRI and CT scan was a drawback of the study, and we tried to solve it by read-back analysis.

## Conclusions

The Games-Howell test revealed that the coronal arc length and sagittal arc length among schizophrenic patients were statistically significantly longer than the healthy control. However, the difference between schizophrenic and healthy control regarding head circumference was marginally significant. Schizophrenic patients had a significantly shorter total facial height and upper facial height than healthy controls. Regarding facial depth, schizophrenic patients had significantly shallow upper, middle, and lower facial depth. This finding gives clues to the neurodevelopmental basis of schizophrenia and could be used as a tool among diagnostic criteria in making an early diagnosis of schizophrenia.

## Supplementary Information

**Additional file 1. **Landmarks on craniofacial region used for anthropometric assessments.

## Data Availability

The dataset supporting the conclusion of this article is available from the authors on request.
