# Supplementary material for: The neurodevelopmental basis of schizophrenia: clinical clues from craniofacial dysmorphology in northwest Ethiopia, 2020
Source: BMC Neurosci. 2021 Sep 29;22:59. doi: 10.1186/s12868-021-00663-y (PMC8480025; doi:10.1186/s12868-021-00663-y)
Supplement: Supplementary file 1 — Additional file 1. Landmarks on craniofacial region used for anthropometric assessments. [file 12868_2021_663_MOESM1_ESM.docx]

**Additional file 1.**

Landmarks

*Vertex*: is the highest point of the head when the head is oriented in the Frankfort Horizontal.

*Glabella* is the most prominent midline point between the eyebrows and is identical to the bony glabella on the frontal bone.

*Opisthocranion* is the most prominent situated in the occipital region of the head and is most distant from the glabella; it is the most posterior point of the line of greatest head length

*Tragion is* the notch on the upper margin of the tragus of the ear

G*nathion* is the lowest median landmark on the lower border of the mandible. It is identified by palpation and is identical to the bony gnathion.

*Trichion* is the point on the hairline in the midline of the forehead

*Glabella* is most prominent midline point between eyebrows. It is identified by palpation and is identical to the bony glabella.

*Nasion* is the point in the midline of both the nasal root and the nasofrontal suture.

*Subnasale is* the midpoint of the angle at the columella base where the lower border of the nasal septum and the surface of the upper lip meet.

*Stomion is* the point at the crossing of the vertical facial midline and the horizontal labial

fissure between gently closed lips, with teeth shut in the natural position.
